# Embracing complexities in agricultural water management through nexus planning

**DOI:** 10.1002/ird.3041

**Published:** 2024-09-29

**Authors:** Mark D. Smith, Alok Sikka, Cuthbert Taguta, Tinashe L. Dirwai, Tafadzwanashe Mabhaudhi

**Affiliations:** 1 International Water Management Institute, Colombo, Sri Lanka; 2 International Water Management Institute, New Delhi, India; 3 Centre for Transformative Agricultural and Food Systems, School of Agricultural, Earth and Environmental Sciences, University of KwaZulu-Natal, Pietermaritzburg, South Africa; 4 International Water Management Institute (IWMI-SA) – Southern Africa Regional Office, Pretoria, South Africa; 5 Centre on Climate Change, London School of Hygiene and Tropical Medicine London, UK; 6 Institute for Water, Environment and Health, United Nations University, Richmond, Ontario, Canada

**Keywords:** equity, livelihoods, nexus, planetary health, policy, resilience, sustainable development, equité, moyens de subsistance, politique, santé planétaire, résistance, développement durable, lien

## Abstract

A major challenge for agricultural water management (AWM) in the 21st century is to feed a growing population in the face of increasing intersectoral resource competition, evolving diets, degradation, pandemics, geopolitical conflicts and climate change. This has to be achieved within the planetary boundaries and without compromising the livelihood and environmental (ecosystem) objectives linked to water, including provisioning, supporting and regulating services. This paper uses a systems and nexus lens to unravel the centrality and complexities in AWM, with particular emphasis on the interconnected dimensions and objectives of AWM, as well as its practices and technologies. AWM exists beyond water and food with linkages to human and environmental well-being. AWM needs to catalyse transformation and integrate approaches across systems, users and scales to meet its objectives in a changing climate. It must provide perspectives beyond productivity, managing water risks and safeguarding food security – as important as these are – and integrate our understanding of the interconnected climate, land, water, food and ecosystems to address planetary health outcomes. By doing so, AWM could catalyse contextualised, equitable, innovative solutions that acknowledge local socio-economic and institutional structures and limitations while catalysing sustainable development and climate resilience.

## Abstract (fr)

L’un des principaux défis de la gestion de l’eau agricole (GEA) au XXI^e^ siècle est de nourrir une population croissante dans un contexte de concurrence intersectorielle croissante pour les ressources, d’évolution des régimes alimentaires, de dégradation, de pandémies, de conflits géopolitiques et de changement climatique. Cet objectif doit être atteint dans les limites de la planète et sans compromettre les objectifs de subsistance et environnementaux (écosystèmes) liés à l’eau, notamment les services d’approvisionnement, de soutien et de règlementation. Cet article utilise une perspective systémique et de liens pour démêler la centralité et les complexités de la GEA, en mettant l’accent sur les dimensions et les objectifs interconnectés de la GEA, ainsi que sur ses pratiques et ses technologies. La GEA existe au-delà de l’eau et de l’alimentation et possède des liens avec le bien-être humain et environnemental. La GEA doit catalyser la transformation et intégrer des approches entre les systèmes, les utilisateurs et les échelles afin d’atteindre ses objectifs dans un climat en évolution. Elle doit offrir des perspectives au-delà de la productivité, de la gestion des risques liés à l’eau et de la sauvegarde de la sécurité alimentaire – aussi importantes soient-elles – et intégrer notre compréhension des liens entre le climat, la terre, l’eau, l’alimentation et les écosystèmes pour répondre aux résultats en matière de santé planétaire. Ce faisant, la GEA pourrait catalyser des solutions contextualisées, équitables et innovantes qui reconnaissent les structures et les limites socio-économiques et institutionnelles locales tout en catalysant le développement durable et la résistance au climat.

## 1 Introduction

Although humanity acknowledges water’s importance and biophysical constraints, the continued poor management of water resources causes grand environmental challenges that undermine the availability and sustainability of water resources in many places (Hogeboom, 2020). Water is a precious resource that sustains all life forms on earth, human-ecological systems, development and the environment (Hogeboom, 2020). For example, freshwater ecosystems sustain almost 10% of all described species on Earth and provide important ecosystem services that support human welfare and livelihoods (Acreman et al., 2020). Despite its importance, freshwater is finite (Hogeboom, 2020; Perrone & Hornberger, 2014), makes less than 0.01% of the world’s water resources and covers approximately 0.8% of the Earth’s surface (Acreman et al., 2020).

In addition to the grand environmental challenges that undermine the availability, accessibility and sustainability of water resources, increasing water demand from all sectors is straining the finite and compromised water resources. The increasing demand for freshwater resources continues to grow due to multiple stresses, such as population growth (United Nations, Department of Economic and Social Affairs, Population Division [UN-DESA], 2022), socio-economic development (Roser, 2021), urbanisation (United Nations Economist Network [UNEN] and UN-DESA, 2020) and changing consumption patterns (Organisation for Economic Cooperation and Development (OECD) and Food and Agriculture Organization of the United Nations (FAO), 2023). Shocks such as climate change, pandemics (e.g., COVID-19) and geopolitical conflicts (e.g., Russia-Ukraine) and their impacts amplify the insecurity of water resources and the realisation of the water-related Sustainable Development Goals (SDG) (Ghorai & Tapia, 2022; United Nations Development Programme [UNDP], 2022).

Projections at the global level indicate that by 2050, approximately 11 and 10% (30% by 2,100) of the current crop- and grasslands, respectively, could be vulnerable to climate-driven water stress that results in loss of some productive capacity, with Africa and the Middle East, China, Europe and Asia, particularly at risk (Caretta et al., 2022; Fitton et al., 2019). Similarly, the future global drop in crop yields due to climate change, especially the projected increases in temperature, shifts in rainfall patterns and elevated surface carbon dioxide concentrations, is estimated to be about 1% per decade, to as high as −24% by 2,100, while increased, potentially concurrent climate extremes will periodically increase simultaneous losses in major food-producing regions (Jägermeyr et al., 2021; Kerr et al., 2022; Müller et al., 2021).

Without adaptation, each degree-Celsius increase in global mean temperature would, on average, reduce global wheat yields by 6.0%, rice by 3.2%, maize by 7.4% and soybean by 3.1% (Zhao et al., 2017). As a result, the business-as-usual future projections for 2030 and 2050 relative to 2012 predict demand increases of 40–100% for energy and 35–70% for food, which will require 20–55% more water and 10–30% more agricultural land (International Renewable Energy Agency (IRENA), 2015; FAO, 2022; United Nations Educational, Scientific and Cultural Organization [UNESCO, 2022). By 2030, the demand for freshwater is expected to exceed the current available supplies by 40% (Weiss & Slobodian, 2014). By 2050, the number of water-sheds facing high year-to-year variability, or less predictable water supplies, is expected to increase by 19%, while the human exposure to clean water scarcity at least one month per year increases to 56–66% by the end of the century (Jones et al., 2024; Kuzma et al., 2023). Relative to 2014, total global water withdrawals for irrigation are projected to increase by 10% by 2050, while global food production (+1.1% annually over the next decade) is projected to continue lagging behind consumption (+1.4% per annum) (FAO, 2018b; World Economic Forum [WEF], 2023). The biggest changes in these future demands for water, energy and food will likely be experienced in Sub-Saharan Africa (SSA) (Kuzma et al., 2023).

Thus, perpetuation of the business-as-usual water management, including that for agriculture, will likely fail to address the crisis, and this emphasises the need for pausing and reflecting on historical and current perspectives and practices of agricultural water management and building on them for informing improvements. Limiting global warming to 1.5 °C would potentially reduce water-related risks across regions and sectors, compared to global warming levels of 2 and 4 °C (Caretta et al., 2022). This perspective proposes novel agricultural water management practices that enhance water security amidst climate change and increasing depletion and degradation. We provide transitional pathways that drive water management from the current linear approaches towards the circular economy.

## 2 Challenges Facing Awm

The grand challenges facing humankind revolve around the water- energy- and food-related triple planetary crisis of biodiversity loss, pollution and climate change (Intergovernmental Panel on Climate Change (IPCC)., 2022). These challenges emanate from anthropogenic activities that have breached two-thirds of the nine planetary boundaries, including land system change, freshwater (blue, green) change and climate change (carbon dioxide concentration, radiative forcing) (Ghorai & Tapia, 2022; Richardson et al., 2023). Water, of which agriculture for food production is the largest consumer, is at the centre of the climate, pollution and biodiversity crisis. From water scarcity and droughts to floods, climate change affects water security, agricultural production, food security, ecosystem integrity and livelihoods (Ghorai & Tapia, 2022; Mirzabaev et al., 2023). This complicates resource security challenges and places the Earth well outside the safe operating space for humanity as the ecological reserve has been breached (Kim & Kotzé, 2021).

Agriculture is the most water-intensive sector, responsible for nearly 72% of fresh water withdrawal, and this emphasises the urgent need for transitioning towards more sustainable agricultural and food-production/consumption systems (Ingrao et al., 2023). Over half (60%) of the world’s irrigated agriculture faces extremely high water stress (Kuzma et al., 2023). The total global irrigated crop area is expected to increase by 12% to 394 Mha (million hectares) by 2030, yet possibilities for expanding irrigation are increasingly under threat (Ringler, 2017; Rosegrant et al., 2009). Livestock production is projected to grow rapidly, and more water is needed due to the increased demand for livestock feed (Rosegrant et al., 2009).

The water demand growth rates are unsustainable across all sectors (International Commission on Irrigation and Drainage [ICID], 2017). For example, water with-drawals between 1960 and 2018 increased by 100% in agriculture (livestock 150%, irrigation 200%), 90–300% in industry and 300–600% in domestic use (Otto & Schleifer, 2020; Pirlea et al., 2023; Ritchie & Roser, 2018). Non-irrigation water consumption is expected to more than double by 2050, and this may trigger the reallocation of water from agriculture to urban centres to meet fresh water needs in growing cities (Rosegrant et al., 2009; UNESCO, 2023). Similarly, agricultural production is threatened by the increasing scarcity and diminishing quality of land and water resources (FAO, 2018a). The challenge is related to expanding irrigated areas, among others, without any further increase in water withdrawals to increase the climate resilience of agriculture (ICID, 2017). These challenges necessitate transitioning to a circular economy in the wastewater sector and promoting wastewater recycling and reuse (Voulvoulis, 2018).

## 3 An Integrated Nexus Planning Perspective For Interlinked Awm Challenges

Agriculture exists in a broader and complex economic, sociocultural, biophysical, ecological and environmental context, and agricultural water (AW) use is embedded in larger systems, including the nested and diverse agroecosystems comprising natural and human systems (Manzungu et al., 2017; Uhlenbrook et al., 2022). AWM is central, pivotal and integral to solving economic, social and environmental issues and tackling poverty and hunger’s most important human concerns (Gundogdu, 2020). The AWM system consists of multiple subsystems at multiple levels with multiple components and dimensions with inherently complex feedback (Zhu et al., 2019) - it briefly presents the diversity of domains, disciplines, actors, objectives, motivations, drivers for change, spatial scales and challenges/barriers that are inherent in the AWM challenge, which is also mirrored in the diversity of interventions to improve the use of AW. The multiple interconnected AWM objectives include, among other things, improving water productivity for producing more nutritious food, income, better livelihoods and ecosystem services with less water (Molden et al., 2010; Zhu et al., 2019). Such objectives are interconnected either synergistically (beneficial) or antagonistically (negatively, trade-offs), and the latter presents the complexity which has to navigate to be navigated (Sušnik et al., 2023). Smith et al. (2023) highlighted some major previous AWM developments, their successful objectives and their negative spillover effects on other AWM-related objectives, dimensions and domains. According to Editorial (2023), access to water and food and their effects on environmental and human health should be assessed and addressed together; hence, confining AWM to isolated sectoral boxes, e.g., water or food, is an insult to its centrality in multiple objectives for the people, prosperity and planet. A common gap in AWM is inadequate understanding of causal relationships in the system (Valencia Cotera et al., 2023). According to Uhlenbrook et al. (2022), addressing the multiple AWM objectives requires integrated approaches and multi-scale and multi-actor thinking based on a good understanding of scale dependency and interdependencies of nested systems. In essence, AWM is not isolated as it is linked to other related and equally important processes.

**Figure 1:**
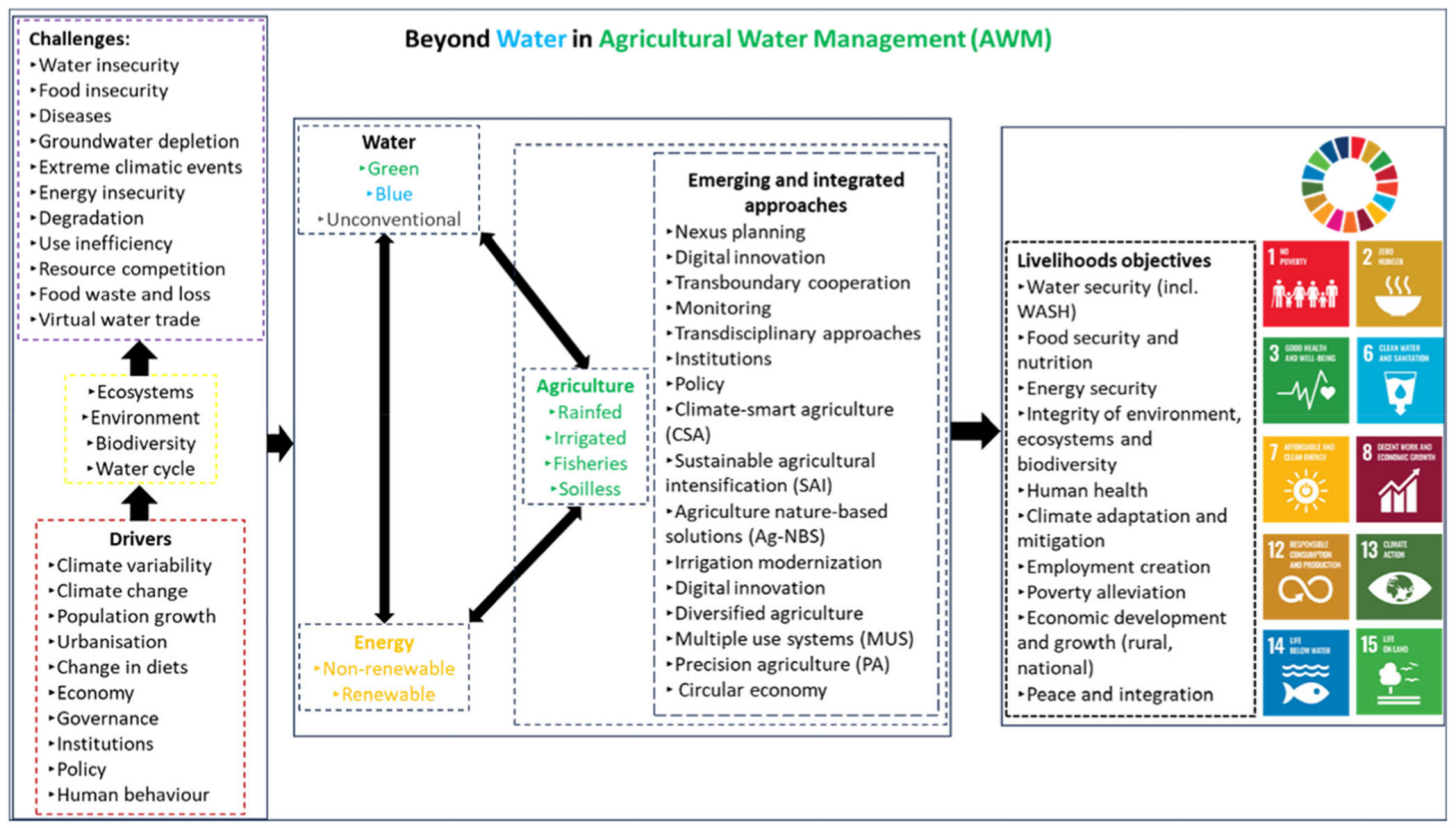
A nexus-based framework linking agricultural water management to other equally important processes.

Similarly, Zhu et al. (2019) proposed using a systems and transformative approach to AWM dissection and analysis in agricultural water management that adequately considers the inter-relationships between human and natural systems. A systems approach to AWM identifies and assesses the roles of multiple actors and identifies significant contextual relationships among the system’s components for understanding and building on connections and feedback loops among relevant actors and practices (United States Agency for International Development [USAID], 2015). Understanding the complex interactions and feedback between AWM subsystems at a variety of scales and under multiple dimensions is a pre-requisite for efficient, productive, profitable and sustainable future irrigated agriculture that produces more crops and higher-value crops with less water and other natural resources without backfiring and opening new problems that might be larger than the original problem that was addressed (Zhu et al., 2019). According to Zhu et al. (2019), this requires avoiding treating AWM subsystems as isolated entities but considering the interconnectedness and interdependencies between AWM and different sectors, systems or resources to ultimately manage the inherently complex relationships at multiple levels while holistically scrutinising the pros and cons of identified AWM interventions beyond their direct effects for the locale they were designed for. Thus, this article uses integrated nexus thinking beyond water to present the centrality of AWM within the complexities of sustainable development while advocating the use of the nexus approach in holistically understanding and improving AWM through consideration and balancing the different objectives and divergent interests of the multiple dimensions, sectors and actors in and linked to AWM (Figure 1).

Such a broad and integrated framework builds on and departs away from the prevailing silo-thinking to account for the interconnections within and between sectors and dimensions. Even the triple planetary crisis of climate change, pollution and biodiversity (nature) loss that AWM currently faces are inextricably interlinked (Hellweg et al., 2023; United Nations Framework Convention on Climate Change [UNFCCC], 2022) and managing these issues/threats in silos can accrue singular objectives with potential for spillovers while the integrated management may simultaneously achieve multiple objectives through synergy and triangulation (Qi & Terton, 2024; Zaman et al., 2024).

Similarly, integrated approaches can be instrumental in meeting the three central challenges of the Anthropocene, which are mitigating and adapting to climate change, protecting biodiversity and ensuring human well-being (Seddon, Chausson, et al., 2020). To achieve a holistic solution to today’s wicked challenges around the complexities of AWM and the sustainability of the present and mid-future food production systems, the think tank space needs to adopt systems-oriented research and decision-making frameworks to guide AWM practices. Solely focusing on disciplinary approaches to AWM hinders cascading benefits derived from multiple-perspective thinking. For example, the evolution of engineered irrigation technologies sought to improve water use efficiency (Horst, 1998); however, during the modernisation era, the engineering designs failed to accommodate the socio-cultural-political norms in the schemes, thus creating a water governance crisis (Dirwai et al., 2019a, 2019b). To avoid perpetuating the ‘same’ crises, embracing a trans-disciplinary methodology must accommodate all stake-holders to co-create solutions to today’s wicked problems. Multi-stakeholder involvement bridges the gap between science, governance and local and indigenous knowledge to bring about enhanced knowledge around the water, food and biodiversity systems across scales.

## 4 Understanding The Centrality Of Awm For Sustainability

There are calls for AWM to go beyond traditional yield increase and stability objectives to maximise benefits and outcomes under growing water scarcity (Ringler, 2021). The impacts of AWM on the broader society, environment and economy can be positive or negative and interconnected depending on the context. With an understanding of the centrality of water, here we highlight the interconnectedness of AWM in diverse livelihood domains, dimensions, sectors and objectives, including resource security, health (human, environmental, ecosystems, biodiversity), climate change and sustainable development.

### 4.1 AWM and food security and nutrition

Whether natural rain or artificial application (irrigation), water is central to producing food and fibre, supporting human activity and socio-economic development (Sušnik et al., 2023; UNESCO, 2024). Agriculture uses nearly 72% of all freshwater (blue water) withdrawals, and about 85% of withdrawn resources are consumed in irrigated agricultural production (Ringler et al., 2023; UNESCO, 2024). Rainfed agriculture produces more than 60% of the world’s cereal grains on 80% of the world’s cropland (FAO, 2021b). Farmers worldwide rely heavily on rainfall for food production, especially in Sub-Saharan Africa (Ringler et al., 2023). Rainfed agriculture generates relatively high yields in areas with reliable rainfall and good soils, such as temperate regions. However, the lack of access to agricultural water reduces the production potential of millions of smallholder farmers dependent on rainfed agriculture (UNESCO, 2024).

Supplemental/life-saving irrigation of rainfed crops with rainwater harvesting stabilises (yields and prices) and boosts yields even higher through reduced reliance on rainfall (Caretta et al., 2022; Molden et al., 2011; Sikka et al., 2022). Despite the dominance of precipitation as the major source of water for the world’s crop production globally, irrigation waters the production of key staple crops (rice, wheat, maize), fruits and vegetables and other crops such as sugarcane and cotton (Ringler, 2021). Full irrigation increases crop yields and cropping intensity, stabilises food production and increases the productivity of other complementary inputs such as high-yielding variety seeds (HYV), fertiliser and labour (Giordano et al., 2023; Ringler, 2021). Irrigated agriculture uses approximately 20% of all cultivated agricultural land to produce 40% of food and fibre and 60% of cereals (FAO, 2022; Ringler et al., 2023). This is why, in many parts of Sub-Saharan Africa, improved AWM can potentially increase rainfed crop yields in the range of double to triple (Domenech & Ringler, 2013; Giordano et al., 2012).

### 4.2 AWM affects water security

The relationship between agriculture and water is two-way. Agricultural production depends on water security and vice versa because water for agriculture is the most water-intensive sector and constitutes a major proportion of total global water withdrawals (nearly 72%) and consumptive water use (~90%) (Ingrao et al., 2023; McDermid et al., 2023). The irrigated area almost doubled during the period 1961–2014 globally (190%) and in Sub-Saharan Africa (250%), respectively (Pellegrini & Fernández, 2018). Given the high water-consumptive nature of agriculture, the expected expansion in total global irrigated crop area (12% by 2030) and rainfed area (9% by 2030) will likely exacerbate water scarcity unless alternative water sources are unlocked (Ringler, 2017). Between a quarter and 40% of the global area equipped for irrigation relies on groundwater. In comparison, the global consumptive use of groundwater in irrigation is estimated to account for 43% of total consumptive irrigation water use. In many cases, surface water and groundwater are used for irrigation areas to supplement the former or dilute the latter (Ringler, 2021; UNESCO, 2024).

Irrigation implemented without considering the synergies and trade-offs with other interlinked sectors has the risk of exacerbating water insecurity by depleting surface water and groundwater. Irrigation has caused a reduction in the annual discharge of some of the world’s major rivers (Rosegrant et al., 2009). According to Mehta et al. (2024), more than half (52%) of the irrigation expansion occurred in areas already water-stressed in 2000, with India alone accounting for 36% of global unsustainable expansion. Reported cases of large-scale depletion of surface water resources due to excessive diversion of water include the shrinkages of inland waters in Asia (Aral Sea, Lake Urmia, Dead Sea, Caspian Sea, Sea of Galilee or Lake Kinneret), Sub-Saharan Africa (Lake Chad), South America (Lake Poopo) and North America (Great Salt Lake, Lake Abert), and the drying up of large rivers such as Colorado (United States), the Krishna (India) and the Yellow (China) (Ringler, 2021; Wine & Laronne, 2020; Zhu et al., 2019). For example, water depletion, salinisation and water pollution in the Indus Basin Irrigation System (IBIS) are attributed to irrigation (Ringler, 2021). Agricultural powerhouses, including India, China and the United States, heavily rely on groundwater for food production, and it is estimated that about 15% to 35% of global irrigation withdrawals are unsustainable, especially from groundwater sources, which are non-renewable or exploited beyond recharge rates (Rosegrant et al., 2009). Groundwater pumping for irrigation has increased dramatically in the past five decades, and a key adverse outcome is groundwater over-extraction (Caretta et al., 2022; Editorial, 2023; Ringler, 2021). Excessive groundwater abstraction causes water scarcity and quality concerns (Ringler, 2021; Rosegrant et al., 2009). Surface water interacts with groundwater by exchanging water (recharge) and solutes (pollution). Thus, water withdrawal from surface sources can deplete groundwater, or conversely, excessive groundwater pumping can deplete water in streams, lakes or wetlands (Winter et al., 1998). For example, groundwater depletion causes decreases in groundwater levels, which reduces subsurface flows (baseflow) in surface water bodies (rivers and streams) that are connected to aquifers, thus reducing runoff and water availability for other users and uses (Fienen & Arshad, 2016; Ringler, 2021).

Similarly, surface water pollution can cause groundwater quality degradation; conversely, groundwater pollution can degrade surface water (Geris et al., 2022; Winter et al., 1998). Thus, the over-abstraction of one or both sources for AWM causes depletion of the other or both sources and undermines water security for agriculture and the broad spectrum of economic and social uses. Reported examples of large-scale groundwater depletion mainly for agricultural purposes include in Asia (south, central), Northwest India, China (North China Plain), the Middle East and North Africa, North America, parts of Australia and many localised areas in southern Europe (Chakraborti et al., 2023; Fienen & Arshad, 2016; Zhu et al., 2019). In irrigated agriculture, the poor economic treatment of irrigation water, whereby the water is either not priced or priced as a low service fee that is detached from the irrigation water use and a small proportion of the true cost value of water, is partly responsible for the inefficient use that is water propagating water scarcity (Perrone & Hornberger, 2014; Ringler, 2021). Water security is compromised when the use of water in agriculture pollutes and contaminates surface water with residues (fertiliser, biocide, sediment, coliform) and trace elements (organic, inorganic), which may require treatment before other uses (Perrone & Hornberger, 2014; Rosegrant et al., 2009).

Due to the intricate linkages between water security and food security, the AWM-induced depletion of water resources in poor countries causes human suffering due to a lack of water and food to meet basic needs. Thus, although agricultural water use is a major contributor to water scarcity in arid and semiarid areas of the world, AWM is pivotal to solving the water crisis because it uses the largest volume of water. Both future water and food security critically depend on proper agricultural water management (AWM) (Zhu et al., 2019).

### AWM and energy security

Water use in agriculture depends on and contributes to energy security. Direct and indirect energy use, among others, contributed to agricultural intensification and increased yields in the recent and far past (Pellegrini & Fernández, 2018). Infrastructure and equipment for AWM, such as water (and wastewater) treatment systems, water pumps, irrigation systems, land preparation implements and postharvest and processing technologies, are powered by direct energy such as human labour, draught power, fuels, wind, solar and electricity. On the other hand, indirect sequestered or embedded energy inputs used in conjunction with AWM include fertilisers, chemical pesticides, insecticides and herbicides (FAO, 2012; Schnepf, 2004; Woods et al., 2010).

As such, the food production and supply chain consumes about 30% of global energy (FAO, 2012, 2018b; Sivakumar, 2021). The lack of electricity access affects water lifting for irrigation (Ringler, 2021). Depending on climatic regions, farming practices, crop type and use of utility power, AWM, especially irrigation pumping, is responsible for the highest portion (15–48%) of direct energy consumption allocated for crop production than other agricultural activities (Qin et al., 2024; Shi et al., 2024; Storm et al., 2018). Similarly, energy represents around 40% of the total water costs (around 40%), and it has claimed its role as a major factor with importance equal to water availability in pressurised systems (Rodríguez-Díaz et al., 2011). Groundwater pumping accounts for 89% of the total energy consumption in irrigation (Qin et al., 2024). In the last decades, the energy embedded in the main oil-based inputs (machinery, fuel and fertilisers) increased globally at a rate slower than crop production, resulting in a recent overall improved energy-use efficiency (EUE) due to advances in irrigation, among others (Pellegrini & Fernández, 2018). In general, at the farm level, the water-inefficient surface irrigation systems are the most energy-efficient, while the pressurised water-efficient systems (sprinklers, drip) are the most energy-intensive (Jamali et al., 2021; Qin et al., 2024; Taguta, Dirwai, et al., 2022). The energy requirements for agricultural wastewater treatment depend on the concentrations of fertiliser, biocide, sediment and coliform residues from crop and livestock production (Perrone & Hornberger, 2014). Meanwhile, groundwater pumping is energy-intensive, especially in irrigated agriculture (Djumaboev et al., 2019; Siddiqi & Wescoat Jr, 2013).

AW, land and produce are sources of energy. Agricultural products such as sugarcane and their residue and waste, such as cow dung, are used as feedstock in bioenergy production, including biofuels (e.g., ethanol, methanol, biodiesel) and biogas. The production of biofuels is highly water and land-intensive depending on crop type, climate, soil conditions and farm practices, among others (Giordano et al., 2023). About 2–7% of global cropland is used, 44 km^3^/year of water is withdrawn, and 16 km^3^/year of water is consumed water for biofuel production (Biofuels International, 2022; Popp et al., 2016; Union zur Förderung von Oel - und Proteinpflanzen E.V. [UFOP], 2021). Irrigation of feedstocks for biofuels uses around 1% of all water withdrawn for agricultural purposes and affects water resources, both water quantity (withdrawals, evapotranspiration) and quality (nutrient loading) (Rosegrant et al., 2009). Agriculture biomass contributes more than 30% of global household energy for heating, cooking and lighting (Saleem, 2022). Bioenergy accounts for around 10% of the world’s primary energy demand today, as solid biomass (90%), liquid bio fuels (7%) or gaseous form (less than 3%). The latter form consists of biogas and biomethane, representing an even smaller 0.3% share of total primary energy (International Energy Agency [IEA], 2020; Kabeyi & Olanrewaju, 2022). AWM developments, such as irrigation development, generally lead to infrastructural improvements, including rural electrification for better access to electricity in homes, with reduced reliance on firewood for cooking (Dube, 2016). For decentralised systems such as solar-based irrigation in remote, underserved rural areas, positive spillover effects include increased household water (livestock watering, domestic uses) and energy security (feed-in to grid, lighting, cooling) (Birhanu et al., 2023; Hartung & Pluschke, 2018).

Multiple-use dams wherein irrigation water is drawn provide water whose kinetic energy moves turbines to generate hydroelectricity. Similarly, the kinetic energy of flowing water in open and closed channels can turn small turbines to produce electricity. On the other hand, solar PV panels can produce energy in co-location with crops/livestock on the same piece of land (agrivoltaics or agro-photovoltaics, APV) or with a surface water body (floating photovoltaics, FPV) or with fish in the water body (aquavoltaics) (Vivar et al., 2024). The shading effects of such co-located water-energy-food systems accrue several benefits, including reduced land requirements, surface water evaporation, plant evapotranspiration, algal blooms and weed growth, while the water-cooled micro-climate improves the generation efficiency of the solar PV panels (UNESCO, 2024). Such integrated multiple-use systems accrue benefits of energy security and increased productivity. Thus, how agricultural water is managed, i.e., AWM, affects how water is allocated between agricultural and energy generation in large dams and how water (dams, reservoirs, channels), energy (turbines, solar PVs) and food systems are co-located all which ultimately determine energy and water security. Thus, in this regard, AWM needs to give equal consideration to energy production and management for sustainable food systems.

### 4.4 AWM and the integrity of the environment, ecosystems and biodiversity

Ecosystems regulate the quantity and quality of water and land available across space and time and its quality in processes such as the water cycle, streamflow, groundwater recharge and atmospheric water recycling (Kerr et al., 2022; UNESCO, 2024). Although agriculture heavily depends on the provisions and services of the environment, the former interacts with the latter in ways that affect ecosystems and biodiversity (Carmona-Moreno et al., 2021; Roidt & de Strasser, 2018). Cultivable agricultural land and the goods and services it provides are finite, irreplaceable and irreproducible resources with limits to expansion (Brain et al., 2023; Ritchie & Roser, 2019). The total global harvested area is estimated at 1.3 billion hectares and comprises 351 Mha and 915 Mha under irrigated and rainfed agriculture, respectively. By 2030, the total global harvested area is expected to expand by approximately 10% (to 1.4 billion hectares), with the highest increase of 12% (to 394 Mha) for the irrigated area compared to the 9% (997 Mha) increase in rainfed area (Ringler, 2017). Yield stability and crop intensity increases in irrigated agriculture reduce the burden of irrigation expansion on important forest resources, limited cultivable agricultural land and marginal lands prone to erosion and degradation (Giordano et al., 2023; Ringler, 2021). On the other hand, land degradation reduces both land productivity and water use efficiency (Rosegrant et al., 2009).

How agricultural water, land and other inputs are used greatly determines land use and land cover, land quality and water quality, with secondary effects on the well-being of the people and the planet (Zhu et al., 2019). However, water-related ecosystems are by far the most heavily impacted by poor land management, over-use of water and land conversion. Irrigation, fertigation and fertilisation imply a contribution of water and nutrients into the soil for crop growth and development (Singh, 2015). However, poor unsustainable irrigation practices, insufficient drainage systems, poor irrigation water quality and excessive use of fertilisers, among others, can cause and exacerbate the twin menace of waterlogging and soil salinisation, especially in arid and semiarid regions (Hellegers et al., 2022; Singh, 2015; Tarolli et al., 2024). Globally and among others, over one billion hectares in about 100 countries are salinised, and 0.3–1.5 Mha of valuable agricultural land are lost annually due to poor drainage, thus reducing crop production potential (Harper et al., 2021; Kumar & Sharma, 2020; Singh, 2022). Poor drainage, waterlogging and salinisation in the rootzone are detrimental to crop growth, crop yields, soil microorganisms, aeration, nutrient uptake and the environment, thus severely threatening the long-term sustainability of irrigated agriculture in arid and semi-arid areas (Hamidov, Kasymov, et al., 2022; Konukcu et al., 2006; Tarolli et al., 2024). The salinisation of rivers threatens biodiversity and compromises the eco-system, goods and services of rivers, wetlands and lakes, as has been the case in the Aral Sea Basin (Central Asia), the Indo-Gangetic Basin (India), Indus Basin (Pakistan), the Yellow River Basin (China), the Euphrates Basin (Syria and Iraq), the Murray-Darling Basin (Australia), South Western Australia and the San Joaquin Valley (United States) (Hamidov, Daedlow, et al., 2022; Harper et al., 2021; Ringler, 2021). For example, of the 514,000 ha of irrigated farmland in Kashkadarya Province (Amudarya River, Uzbekistan), about 45% of irrigated lands are salinised to some degree and the groundwater (on average 2–3 m below the surface) is also significantly salinised (Djumaboev et al., 2017; Hamidov, Kasymov, et al., 2022). Also, poorly drained waterlogged lands are breeding hotspots of harmful organisms that compromise human health (Singh, 2020). Waterlogging and salinisation also contaminate groundwater (Mohanavelu et al., 2021).

Surface water and groundwater depletion cause environmental degradation (Zhu et al., 2019). Excessive diversion of flows from rivers for agriculture has contributed to the desiccation and severe shrinkage of strategic water bodies with lasting consequences on environmental provisioning services (Ringler, 2021). While water storage and diversion and irrigation infrastructure (e.g., canals) enhance the control of irrigation water for improved AWM, these artificial infrastructures alter flow regimes and the geomorphology of the river and the vegetation in rivers and on its banks, all of which deprive the naturally occurring environmental and ecosystem services provided by free-flowing rivers, streams and lakes (Hellegers et al., 2022). Flow volume degradation was reported in the Aral Sea due to irrigation, among other things (Hamidov, Daedlow, et al., 2022; Hamidov, Kasymov, et al., 2022). Consequences of disrupted natural storage and flows include habitat loss and destruction of resident flora and fauna. In Zimbabwe, the construction of the Tugwi-Mukosi Dam displaced some animals and escalated their conflict with people (Mujere & Chanza, 2022). According to Ringler et al. (2023), more than half of all natural wetland areas have been lost due to human activity, including irrigation, since 1900, and forest degradation affects streamflow regulation. In the Indus Basin Irrigation System (IBIS), irrigation is partly to blame for the near-extinction of the Indus River dolphin, the fragmentation of river ecologies and the destruction of coastal mangrove habitats (Ringler, 2021). The inundation of biomass in water bodies may cause net emissions of greenhouse gases (e.g., carbon dioxide, methane), which may cause localised air pollution problems, global warming and climate change (Giordano et al., 2023).

Similarly, groundwater over-abstraction and overex-ploitation are causing saltwater intrusion and rendering the aquifers unusable due to the salinisation of groundwater (Harper et al., 2021; OECD, 2016; Rosegrant et al., 2009). Water depletion from the over-extraction of groundwater is a major contributor to land subsidence and soil compaction, as reported in San Joaquin Valley (California) and Mekong Delta (Vietnam), which causes damage to infrastructure, such as buildings, roads and bridges, increases flood risk and long-term reduction in groundwater storage (Giordano et al., 2023; OECD, 2016; Ringler, 2021). Receding groundwater tables in China, India, and elsewhere have caused farm income losses, health risks from arsenic to fluorosis and dried-up springs and small streams (Rosegrant et al., 2009).

### 4.5 AWM and human health

Irrigation can positively and negatively affect human health (Zhu et al., 2019). Irrigation enhances nutrition by providing more and more nutrient-dense food to more people through increased crop productivity and the production of a larger variety of crops (Giordano et al., 2023; Zhu et al., 2019). Similarly, the disposable income accrued from agriculture, especially irrigation, may incentivise expenditure in human health care and increase expenditures towards diverse and nutritious foods (Giordano et al., 2023). Thus, AWM can enhance nutrition and health outcomes (Ringler, 2017).

On the other hand, the key environmental challenges of AWM (e.g., water depletion, pollution, contamination) and their impacts on water-based ecosystems and land resources compromise human health and wellbeing (Ringler, 2021). The negative health effects are mainly attributed to the poor quality of irrigation water and the use of chemicals such as pesticides due to improper management (Ringler, 2017). Irrigation water in storage or slow flow and rises in groundwater level can host disease vectors, such as Anopheles mosquitos and snails, which increase incidences of malaria, dengue and schistosomiasis (bilharzia) and can carry bacteria responsible for cholera, diarrhoea, dysentery and other diseases (Giordano et al., 2023; Ringler, 2021; Zhu et al., 2019). Other diseases that can accrue from ill-managed irrigation practices include sleeping sickness (trypanosomiasis). In Zimbabwe, the construction of the Tugwi-Mukosi Dam introduced new disease ecologies such as schistosomiasis, malaria and water-borne diseases in the area, with likeness of seismic earth tremors, while planned developments such as the game reserve will potentially trigger human-wildlife conflicts (Mujere & Chanza, 2022). Wastewater irrigation, especially with untreated or minimally treated wastewater, can cause other ailments in producers (farmers) and consumers due to increased exposure to pathogens (Mora et al., 2022). The contamination of irrigation water, either naturally (e.g., arsenic) or artificially (e.g., pesticides), can lead to health complications for producers (farmers) and consumers. Irrigated agriculture tends to encourage the use of agricultural inputs (e.g., fertiliser, pesticides, herbicides), which increase the exposure to both producers (farmers) and consumers (Giordano et al., 2023). Excessive nitrogen (N) and phosphorus (P) from fertiliser and animal waste in water bodies can contribute to blue baby syndrome and infant illness.

Similarly, the land irrigated with polluted irrigation water is rapidly growing, especially irrigation with untreated wastewater, leading to contamination of drinking water sources, the spread of diseases and millions of cases of illness and thousands of deaths every year (Ringler, 2021; Zhu et al., 2019). The irrigation-induced desiccation and severe shrinkage of large water bodies cause the deterioration of human health, for example, in the case of the Aral Sea wherein cases of higher rates of asthma, cancer and increased infant mortality among residents due to environmental pollution have been reported (Hamidov, Kasymov, et al., 2022; Ringler, 2021). Thus, the management of AW can enhance human health by maximising food security and nutrition, minimising the incidence of pathogens in irrigation water and producing diseases in producers (farmers) and consumers.

### 4.6 AWM, integration, cooperation and peace

Water is shared naturally in the hydrological cycle or spatially in transboundary sources (basins, rivers, lakes, aquifers), which account for 60% of the world’s freshwater flows. In comparison, 88% of Sub-Saharan African countries share a transboundary basin, and the African continent has the highest proportion of transboundary basins, covering an estimated 64% of the land area (Sušnik et al., 2023; UNESCO, 2024). Water resources are mobile, flow across artificial borders and usually have upstream and downstream users who often have conflicting needs, such as consumption, withdrawal or diversion, with others using the water in situ (Almulla et al., 2018; Perrone & Hornberger, 2014). Water insecurity, insufficient quantity and poor quality can be major drivers of conflicts and displacement, particularly in regions where water resources are limited or unevenly distributed and riparian states distrust one another and use water unilaterally (UNESCO, 2024). According to the Water Conflict Chronology database (https://www.worldwater.org/water-conflict/), water-related conflicts manifest in three forms based on water’s use, impact or effect within the conflict. Water as a trigger or root cause of conflict is a dispute over controlling water or water systems or where economic or physical access to water or water scarcity triggers violence. Water as a weapon of conflict is where water resources, or water systems themselves, are used as a tool or weapon in a violent conflict. Water resources or water systems as a casualty of conflict is where water resources or water systems are intentional or incidental casualties or targets of violence (Pacific Institute, 2023). In some local and international water-related conflicts, at least one involved party uses the shared water resources for agriculture, such as irrigation. In contrast, other contested uses include hydropower generation and domestic. AWM assets and infrastructure damaged as casualties in water-related conflicts include dams, canals, pipelines, wells, springs, fields, grazing land, crops and livestock, while some led to injuries and casualties. From 2000 to 2023, intra-country conflicts dominated (85.2%) the water-related confrontations, followed by inter-country (14.8%).

The water crisis and related conflicts are ranked among the top severe global risks now and in the future. New economic and social realities, such as climate change and geopolitical changes, prevail and affect water resources (UNESCO, 2024; World Economic Forum, 2024). Water deficits exacerbated global migration by a 10% increase between 1970 and 2000 (UNESCO, 2024), and such conflicts are key drivers of food insecurity (Food Security Information Network [FSIN] & Global Network Against Food Crises, 2024). Meanwhile, a reinforcing feedback loop links peace and sustainable development because the latter provides the pathway to peaceful societies (United Nations General Assembly [UNGA], 2015). AWM must not perpetuate water-related conflicts but promote prosperity and peace, which are prerequisites for sustainable development (UNESCO, 2024). This can be achieved by treating and using AWM as a trigger, weapon and victim of prosperity and peace to provide common livelihood, development and cost-sharing opportunities that likely exceed those generated by unilateral action. Cooperation over transboundary water resources can multiply economic, social, environmental and political benefits that deliver peace and prosperity at local, national, regional and global levels (UNESCO, 2024). Thus, for securing WEF resources, preserving human life and integrity, regional integration and international peace, parties sharing water for agriculture and other uses must collaborate and cooperate in AWM.

### 4.7 AWM and the sustainable development agenda

AWM is embedded in key global frameworks and blue-prints for sustainable development, including the ‘Future We Want’, an agreed declaration and perspective of the members of the United Nations (UN); and Agenda 2030 for Sustainable Development, a global policy priority and commitment with 17 sustainable development goals (SDG) and 169 targets (Kerr et al., 2022; UN, 2012; UNGA, 2015). Several reaffirmations of the ‘Future We Want’ directly and indirectly speak to AWM through themes on agriculture, food, water, energy, land, soil, environment, ecosystem and rural development, among others (UN, 2012). SDGs are highly interconnected, integrated and indivisible in their quest to balance the three dimensions of sustainable development: the economic, social and environmental, which form the triple bottom line of the people, planet and prosperity (UNGA, 2015). Achieving water-related SGD6 targets is a precondition that potentially enables meeting targets in other SDGs such as for energy production (SDG7), food security (SDG2), climate action (SDG13) and sustainable use of land (SDG15), although the governance of other SDG related resources can feed back to influence the water SDG (Gundogdu, 2020; Mugagga & Nabaasa, 2016; Sušnik et al., 2023). However, water is broad and includes AWM. Here, we narrow the focus to emphasising the centrality of AWM to achieving SDGs, using key AWM thematic components such as water, agriculture, food and soil.

A rapid search in the UNGA Resolution 70/1 ‘Transforming Our World: The 2030 Agenda for Sustainable Development’ shows the explicit mention and reference of water in SDG3 (good health and well-being; Targets 3.3, 3.9), SDG6 (clean water and sanitation; 6.1, 6.3, 6.4, 6.5, 6.6, 6. a, 6. b), SDG11 (sustainable communities and cities; Target 11.5), SDG12 (responsible consumption and production; Target 12.4) and SDG15 (life on land; Targets 15.1, 15.8). On the other hand, agriculture and food are explicitly mentioned and referred to in SDG2 (zero hunger; Targets 2.1, 2.3, 2.4, 2.a, 2.b, 2.c), and SDG12 (responsible consumption and production; Target 12.3), while soil is in SDG2 (zero hunger; Targets 2.4), SDG3 (good health and well-being; Targets 3.9), SDG12 (responsible consumption and production; Target 12.4), SDG15 (life on land; Targets 15.3) (UNGA, 2015). However, a deeper analysis of literature (Sections 4.1 – 4.7; UNGA Resolution 70/1 ‘Transforming Our World: The 2030 Agenda for Sustainable Development’) shows that AWM and its components are explicitly and implicitly mentioned in and thus directly and indirectly linked to almost all SDGs and some of their targets (Gundogdu, 2020).

While AWM is central to achieving some SDGs, some are also pivotal in improving AWM. Investments in sustainable irrigation expansion (SDG Targets 2.4 and 6.4) may contribute to alleviating poverty (SDG Targets 1.1), improving food security (SDG Targets 2.1 and 2.2), ensuring adequate water resources for humans and ecosystems (SDG Target 6.4), increasing resilience and adaptive capacity to climate change (SDG Target 13.1) and halting biodiversity loss (SDG Target 15.1 to 15.5) (Rosa, 2022).

Increasing water use efficiency and ensuring sustainable withdrawals and supply of freshwater (SDG target 6.4), implementing integrated water resources management (SDG target 6.4) and ensuring universal access to affordable, reliable and modern energy services (SDG target 7.1) are central to improving AWM. On the other hand, improved AWM potentially contributes to enhancing agricultural productivity and incomes (SDG targets 2.3, 2.4), ending hunger, achieving food security and improved nutrition (SDG targets 2.1, 2.2) and promoting sustainable agriculture (SDG target 2.4). Similarly, some SDGs are mutually related to AWM because bi-directional feedback loops exist between AWM and the SDGs. For example, improving water quality by reducing pollution, eliminating dumping and minimising the release of hazardous chemicals and materials (SDG target 6.3) and climate action (SDG target 13.2) contribute to improved AWM, which in turn drives protection and restoration of water-related ecosystems (SDG target 6.6) and strengthening resilience and adaptive capacity to climate-related hazards (SDG target 13.1). AWM is linked to SDG1 (no poverty) through SDG6 (clean water and sanitation) because reducing poverty improves adaptive capacity according to the Paris Agreement adaptation goals. The same can be said between SDG1 (no poverty) and SDG2 (zero hunger), the latter whose achievement requires access to adequate water for agriculture SDG6 (clean water and sanitation).

Similarly, AWM, through access to basic infrastructure for SDG6 (clean water and sanitation), promotes achieving SDG3 (health and well-being), while SDG7 (affordable and clean energy) may compete with AW for water for energy production (e.g., hydropower) (Caretta et al., 2022; Kerr et al., 2022). Thus, water resources are central to sustainable development and systems transitions towards climate-resilient development (Caretta et al., 2022; Kerr et al., 2022), while AWM and SDGs and their mutual targets are mutual (Li & Wu, 2024). Similarly, a robust developmental agenda depends on recognising that AWM and its effects cannot be limited to agricultural production only. By narrowing its focus around particular scales and specific decision spaces (agriculture), the world will lose the multiple development trajectories and benefits emanating from holistic and multi-sectoral approaches.

### 4.8 Summary of the centrality of AWM

Several, if not all, of the above seven dimensions of AWM are usually highly intertwined. Neglecting the multidimensional nature of AWM may render an incomplete picture and weaken the development and implementation of AWM interventions (Zhu et al., 2019). The intricate and complex interconnectedness of the individual nodes (energy, food, land use and climate) further assert the centrality of AWM as a driver to sustainable (equitable, just, inclusive) and resilient food systems that acknowledge synergies and trade-offs between AWM and the sustainable and resilient food systems. Holistically transforming agri-food systems encompasses the effective utility of the blue, green and grey water footprint. Climate change threatens agri-food systems; thus, fit-for-purpose resilience is needed to encompass all the moving components involved in AWM. For example, in farmer-led irrigation development (FLID), production pathways, effective management of green water involves understanding the hydrology of irrigation from plant scale to river basin scale. The implication is that best management practices in conservation agricultural (CA) practices promote soil water holding capacity and moisture residence time, thus minimising bare soil evaporation and irrigation frequencies. Sikka (2021) explores how CA can address the challenge of harmonising the synergy among water, energy and food though WEF ‘nexus gains’ as an integrated solution to conserve water, improve soil health, reduce mitigation, reduce the cost of cultivation and preserve ecology in the context of groundwater irrigated rice/cereal-based cropping systems in India. Similarly, Sikka et al. (2022) demonstrated the considerable scope of AWM interventions to improve agricultural resilience to climate change through increased land and water use efficiency, water and energy savings and improved water productivity. The prioritisation of a location and context-specific portfolio of smart AWM practices to make the right investment decisions is emphasised (Sikka et al., 2022).

**Figure 2:**
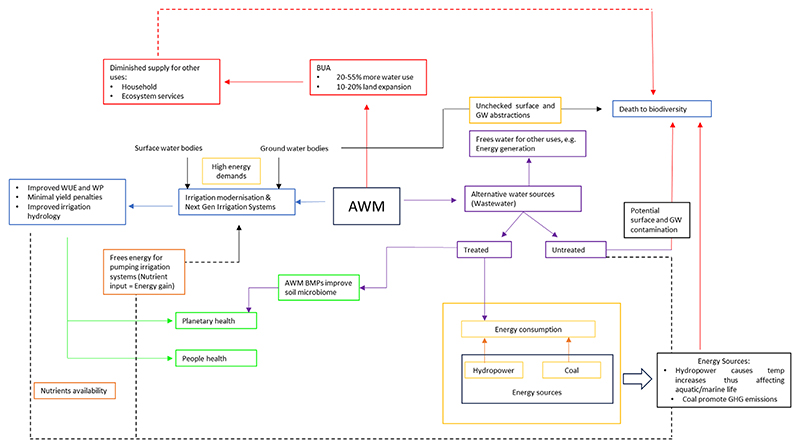
A simplified schematic showing the many levers connected to AWM and simplified pathways that lead to sustainability.

These minute actions can result in aggregated gains at the water source (river basin) by minimising energy requirements for running irrigation pumps, thus availing water for other strategic uses. Although, often, the aggregated benefit can be nullified by land expansion because ‘freed-up’ increases water availability and adequacy (excess at peak demand), prompting farmers to expand irrigated lands, thus making it difficult to realise the water productivity and water use efficiency gains at river basin scale (Lankford & Scott, 2023). This impacts water availability for ecological services (Healthy Planet). Such an example highlights one of the many complexities around AWM and the need to rethink water use across different farming typologies and scales. Figure 2 shows a simplified AWM interconnection with various water footprints and its various demands.

From an ecological perspective, greywater is considered a sustainable pathway to support food production in urban and peri-urban areas in African cities (African Union Commission (AU), 2020; Alliance for a Green Revolution in Africa (AGRA), 2020). The economic outlook is that the African vegetable production value chain is a US$ 200 billion industry (AGRA, 2020). Henceforth, a need exists to set up formal institutional arrangements that facilitate monitored wastewater usage in the respective cities. In the same breadth, setting up people- and planet-centred governance arrangements for wastewater utility is fundamental for growth through easing pressure demands on freshwater sources and protecting human health. The circularity approach (recovery, reuse and recycling) is key to transforming food systems by promoting nutrition and health. Wastewater irrigation has nutrients that industries and irrigation systems could have otherwise manufactured and pumped, respectively, thus touching on the energy component. Water quality negatively and positively impacts ecosystem integrity. Wastewater utility can affect soil quality by potentially altering the DNA of resident microbiomes in the rhizosphere. Soil microbiomes influence how plant roots absorb symbiotic nutrients and discard pathogens (Zolti et al., 2019); thus, greywater use as an AWM strategy is intrinsically linked to ecosystem/planetary health.

## 5 Transdisciplinarity In Awm

AWM is a complex agenda that cannot be addressed by a single sector or actor (International Fund for Agricultural Development [IFAD], 2015). AWM is at the heart and intersection of many disciplines and sectors, including water, energy, agriculture/food, irrigation, climate, environment and economy (Section 4.1-4.7). For example, at its multiple scales, actors with a stake in AWM include farmers, breeders, academia, private sector (inputs, outputs, equipment), non-governmental organisations (NGO), civil society organisations (CSO), extension workers, water users associations (WUA), irrigation experts, basin management authorities, government authorities (local, provincial, national) and line ministries, cities or municipalities, policymakers (environment, agriculture and water), trade unions, development partners (development financial institutions, donors) and international development organisations (Uhlenbrook et al., 2022). These actors have different forms and levels of knowledge and expertise, including technical, social and indigenous knowledge, and they have different roles, including those of public, private and voluntary players.

In the academic space, the main disciplines involved at the different scales of AWM can be categorised as agricultural sciences (plant sciences, biotechnology, agronomy), environmental sciences (soil sciences, ecology), engineering (agricultural, irrigation), water resources management (hydrology, hydrogeology) and social sciences (economics, political sciences, sociology, behavioural sciences, law). AWM involves multiple actors from different sectors with multiple and sometimes different and divergent interests and objectives for people (livelihoods), the planet (environment, ecosystems, biodiversity) and prosperity (economy and lifestyles). According to Uhlenbrook et al. (2022), the wide range of objectives across actors and sectors at the different scales of AWM include optimisation (biomass production, harvestable yield, economic returns, multiple water use [irrigation, WASH, livestock], irrigation system performance, overall water use); reducing cost and labour; serving members of irrigation schemes; balancing water allocation to agriculture and other sectors (e.g., environmental requirements); preventing water related conflicts; climate resilience; achieving food security or self-sufficiency; macro-economic and environmental sustainability; implementation of trade objectives; transboundary cooperation; and political stability. For example, within an irrigation scheme, water managers strive to optimise water use (Uhlenbrook et al., 2022), farmers want to improve their crop (or livestock) yields, quality and income or grow a new crop type (prosperity) (Wahlin & Zimbelman, 2014), NGOs seek environmental conservation and sustainability (planet), the private sector (financial institutions, suppliers) seek to maximise revenues and profits (prosperity), the government seek job creation, foreign currency from exports and food security (people), while political actors are interested in furthering political mileage, securing the farmers’ support and votes in the next elections (Badiani-Magnusson & Jessoe, 2019; Bellmann, 2019; Uhlenbrook et al., 2022).

Thus, AWM is the theatre of interconnected sectors (water, energy, food, etc), disciplines, actors, interests and objectives across a large span of temporal and spatial scales, which require transdisciplinary practice and research through coordination, collaboration, negotiation, leveraging complementary resources and integration of complementary knowledge, expertise, skills and experience (Carmona-Moreno et al., 2021; Uhlenbrook et al., 2022). Transdisciplinarity involves integrating knowledge via engaging non-scientific actors in specific scientific discourses and research questions, e.g. through participatory methods (Daedlow et al., 2016; Hamidov, Daedlow, et al., 2022). This is critical for availing the diverse skills analysts require to understand dynamics involving multiple sectors and resources beyond a purely physical systems analysis (De Strasser et al., 2016). Such transdisciplinary approaches are necessary to collectively balance the multiple priorities, interests and objectives, optimise synergies and harmonise activities and policies while minimising trade-offs and mitigating risks of maladaptation. Similarly, transdisciplinary approaches in AWM reflect the local context and better consider local socio-economic, governance, institutional and technological constraints, thus allowing for contextualised and bespoke scalable AWM practices and innovations (Smith et al., 2023).

Addressing the complex AWM-related challenges requires increased collaboration and partnership between research and knowledge communities to expand the knowledge base and its access (IFAD, 2015). Similarly, cross-fertilisation can be enhanced by expanding the research and development partnerships through diversifying relations to include relevant stakeholders, including government (e.g., parliamentary commissions), industry players, civil society organisations, unions, chambers of commerce and other development agencies such as the private sector (industry players, insurance companies) and major foundations (IFAD, 2015; Pingali, 2012). Such public-private partnership, coupled with research consortia and knowledge exchange platforms (e.g., the Global WEF Nexus Community^1^), is key to scaling up innovative AWM practices through collaboration and co-creation (IFAD, 2015; Uhlenbrook et al., 2022). Communities of science, policy and practice for AWM enable researchers to identify better the knowledge needs of different stake-holders in the most appropriate, relevant and understandable forms (Seddon, Daniels, et al., 2020). Citizen science can simultaneously address data collection and public participation in AWM (UNESCO, 2024). Transdisciplinarity facilitates identifying and reducing uncertainties, biases and limitations and using local knowledge for improved and shared outcomes (McDermid et al., 2023; Sonneveld et al., 2018).

### 5.1 Appraising AWM from an integrated nexus perspective for unpacking and untangling the complexity

Agriculture is a cross-scale example of the WEF nexus concept and problems (Hamidov, Daedlow, et al., 2022). It requires analysis of water, energy use and crop yield interactions in an integrated approach, rather than individually, to optimise the overall resource-use efficiency (Cremades et al., 2016; Shrestha et al., 2015; Smith et al., 2023). Although Djumaboev et al. (2019) reported that quantifying the nexus trade-offs between different sectoral areas has been limited, remarkable progress has been made in appraising AWM from an integrated nexus perspective across scales. Hendy et al. (2024) used an integrated index-based WEF nexus approach and sustainability polygons to evaluate the WEF nexus of producing tomato and melon crops under different management practices, including regulated deficit irrigation treatments and mulching in the Mediterranean region (Egypt and Southern Italy). A similar index-based approach was used to assess and search for actions to improve the WEF nexus of the agricultural sector at a national scale in Egypt (El Gafy et al., 2017a, 2017b). Sadeghi et al. (2020) developed and applied a WEF nexus index to determine the optimal crop plan and agricultural management pattern under irrigated and rainfed agriculture at a watershed scale in Shazand watershed, Markazi Province, Iran. Chakraborti et al. (2023) developed and applied a crop-switching optimisation model for cereals to maximise calorie production and farmers’ profits and minimise water and energy consumption in the IGP. In the same study, they compared the performance of switching crops and irrigation modernisation (from flood to drip irrigation) concerning reduction in groundwater depletion and energy savings (Chakraborti et al., 2023). Taguta, Dirwai, et al. (2022) used radar charts/spider diagrams/sustain-ability polygons in a systematic review and meta-analysis to assess cereal crops’ global water and energy productivities and the water-energy-food (WEF) nexus trade-offs from irrigation modernisation in the production of cereals.

Daccache et al. (2014) used an integrated framework combining a gridded water balance model with a geodatabase and GIS to assess irrigated production’s water demand, energy footprint and irrigation modernisation in the 21 countries surrounding the Mediterranean Sea, Portugal and Jordan. By adapting the methodology by Daccache et al. (2014), Djumaboev et al. (2019) applied a nexus-based quantitative accounting method including CROPWAT 8 to inform water-, energy- and climate-smart optimal planning deficit irrigation schedule at a sub-basin scale in irrigated areas of the Karshi Steppe of Central Asia that are supplied by pumping water uphill (lift-irrigated) from the underlying river. Almulla et al. (2020) used an integrated nexus-based modelling frame-work with climate, land use, energy and water strategies (CLEW), United Nations Economic Commission for Europe (UNECE), Transboundary River Basins Nexus Approach (TRBNA), Geographic Information Systems (GIS) and remote sensing techniques to inform agriculture-water-energy nexus planning (identify the location and extent of irrigated cropland, estimate water and electricity requirements for groundwater irrigation, evaluate selected supply options to meet the electricity demand and suggest the least cost configuration) in the transboundary North Western Sahara Aquifer System (NWSAS: Algeria, Libya, Tunisia).

Li et al. (2019) developed the Agricultural Water-Energy-Food Sustainable Management (AWEFSM) model by integrating multi-objective programming, non-linear programming and intuitionistic fuzzy numbers. The AWEFSM model was applied to analyse quantitatively, under different scenarios at a river basin scale in northwest China, the interrelationships and trade-offs among system components, including water supply demand, energy supply demand, land demand, food production and water and energy footprints. The Farm Performance Calculator (FPC) was developed and applied to conduct energy, economic and environmental analysis based on a simplified evaluation and analysis of the WEF nexus at the farm level in Central Italy (Fabiani, Vanino, Napoli, & Nino, 2020). Fabiani, Vanino, Napoli, Zajíček, et al. (2020) applied the FPC to assess the environmental and economic sustainability of Variable Rate Technology (VRT) nutrient management in Greece and the Czech Republic. Shrestha et al. (2015) used a WEF nexus approach to evaluate the performance of groundwater-based deep tube well and shallow tube well irrigation systems of Sarlahi District (Nepal) in terms of water supply, agricultural output, energy use and management operation and maintenance cost and benefit–cost ratio per unit of irrigated area. Ramirez et al. (2021) used GIS-based tools to capture the systems’ spatial dimension, enabling them to match wastewater supply and water demand points, identify demand hotspots and evaluate techno-economically viable wastewater treatment options in the North Western Sahara aquifer system. These cases present a case and potential for planning, appraisal and implementation of AWM according to the local context of challenges and priorities from a nexus planning perspective. Two powerful tools that feature in several nexus analytic models and can be used across scales to uncover, understand and visualise synergies and trade-offs in AWM are indices and sustainability polygons/radar charts/spider diagrams (FAO, 2021a, 2021c; Smith et al., 2023; Taguta et al., 2023; Taguta, Dirwai, et al., 2022).

Although the WEF nexus was successful in dissecting the WEF interconnections and interactions in the AWM case studies presented above, a quick internet search of nexus-based tools and frameworks that were applied shows that some of the novel ones are not readily available in the public domain for interested users (Taguta, Senzanje, et al., 2022). Similarly, the WEF nexus approach is criticised for neglecting and/or failing to explicitly mention other dimensions such as biodiversity and health (Sušnik & Staddon, 2022) and being non-people-centric, i.e., neglecting the socio-political cultural dynamics around WEF resources utility (Jalonen et al., 2022). The nexus in agriculture goes beyond WEF, and it includes the economic (cost, revenue), socio-economic, ecosystem, climate (greenhouse gas emissions, carbon footprint), environmental (resource use, pollution) and health dimensions, which share some forward and backward loops with agriculture (Avellán et al., 2018; Smith et al., 2023). Hence, the issues of representation are critical in managing WEF resources, and they significantly influence the desired outcomes. Thus, evidence calls for developing WEF nexus frameworks and driving people and planet-centred WEF nexus dialogues that shift focus on disciplinary thinking. For example, improved WEF nexus frameworks and tools can be used to investigate the linkages between the water, energy and food sectors concerning the rebound effects (Hamidov, Kasymov, et al., 2022). In this way, the nexus approach becomes useful in supporting the identification and development of solutions that benefit multiple objectives that may have trade-offs (FAO, 2018b).

### 5.2 The WEF nexus approach for improving AWM governance: integrated coherent policy and strong institutions

AWM goes beyond the use of agricultural water in food and fibre production; it transcends and spans multiple sectors, each with its actors and policies, and thus, its policies and institutions are interconnected to other sectoral policies (IFAD, 2015). For example, AWM policies, such as those for food self-sufficiency, are rarely confined to water, agriculture and food. Still, their implementation transcends other objectives such as water quality, quantity, land, ecosystems, biodiversity, trade, government, the environment and potentially green energy (Sušnik et al., 2023). However, AWM-related policies and institutions have remained vertically and horizontally fragmented, compartmentalised and disconnected due to historical and conventional thinking and responsibilities among governmental authorities and non-state institutions, and this has partly contributed to the prevailing challenges in AWM such as setting conflicting incentives which create imbalance and duplication, and transfer challenges and problems across sectors (Adom et al., 2022; Mpandeli et al., 2018; Pahl-Wostl et al., 2021). Such monocentric and linear approaches in resource management in AWM-related policies promote divergent strategies and entrench silos, which often result in negative trade-offs such as disproportionate resource distribution, which exacerbate inequalities, vulnerabilities and tensions (Mpandeli et al., 2020).

In many cases, the formulation of policies across agriculture, water, land, energy and environment lacks cross-sectoral cooperation and sufficient consideration of their interrelationships or their unintended cross-sectoral consequences (Kanyepi et al., 2024; OECD, 2010; Simelton et al., 2021). This may be due to overlaps in institutional mandates, resource sectoral governance peculiarities, lack of compatibility of scales, differences in policy or enforcement culture, lack of consistency in regulations, and even power imbalances (De Strasser et al., 2016; UNECE, 2015). Policy coherence involves aligning and streamlining incentives and signals of different policies to target groups for minimum conflicts, mainly achieved through coordination and integration between government agencies and ministries (UNECE, 2015).

## 6 Conclusion and Recommendations

We explored the complexities around AWM and how understanding its role from a transdisciplinary lens could help to navigate its challenges. AWM currently faces several challenges that are projected to be exacerbated in the future, including population growth, rapid urbanisation and climate change. Although some of these challenges are interlinked due to their common drivers, AWM-related policy and interventions remain sectoral, and this will likely worsen the crisis due to inherent feedback loops. AWM is a central driver and enabler to multiple livelihood objectives, including social, economic and environmental outcomes, and therefore should move beyond saving water and producing ‘more crops per drop’. Some AWM objectives and dimensions have synergies and trade-offs that need careful management through integrated approaches.

Few case studies have used integrated approaches such as nexus planning to unpack interconnections between AWM-related resources to inform policy and management strategies. Integrated AWM solutions show promise in addressing AWM challenges and mitigating trade-offs across systems and scales. AWM in shared contexts such as transboundary basins can facilitate peace, integration and regional development through cooperation and agreements built on trust and political will among riparian states and adherence to international legal frameworks. However, there is a need to: i) gather evidence on the effectiveness, sustainability and viability of integrated AWM practices and technologies in different regions, especially the contexts of resource-constrained global South, to guide the selection of the most appropriate; ii) develop and test improved integrated data-driven nexus based AWM analytic tools such as frameworks, methodologies and models to assist in testing and implementing AWM practices and technologies; iii) foster transdisciplinary approaches to AWM in research, policy and practice, including the public and private sector, breeders and water experts, as well as between the global South and global North; iv) building capacity and collaborations in collection and processing of alternative sources of data.

Future research will need to: i) gather evidence on the effectiveness, sustainability and viability of integrated AWM practices and technologies in different contexts, such as the resource-constrained Global South; ii) develop and test improved integrated data-driven nexus-based AWM analytic tools that guide implementation of AWM practices and technologies; iii) foster transdisciplinary approaches to AWM in research, policy and practice; iv) build capacity and collaborations in AWM. Improved nexus-based AWM analytic tools must be comprehensive, readily available, and able to analyse policy, SDGs and farmer decisions.

Recognising and locating AWM within the socioecological and socio-economic systems and their challenges, such as the triple planetary crises, provides a solid basis for devising comprehensive strategies that address the interconnections and promote sustainable solutions for the people, planet and prosperity. Improved AWM that safeguards water resources, mitigates pollution and preserves aquatic ecosystems can foster resilience, adaptability and a more sustainable future for human and environmental well-being.

## Data Availability

The data that support the findings of this study are available from the corresponding author upon reasonable request.

## EndNotes

^1^ https://www.linkedin.com/groups/9530027/
